# CXCL12 promotes the crossing of retinal ganglion cell axons at the optic chiasm

**DOI:** 10.1242/dev.202446

**Published:** 2024-01-12

**Authors:** Viet-Hang Le, Clarisse Orniacki, Verónica Murcia-Belmonte, Laura Denti, Dagmar Schütz, Ralf Stumm, Christiana Ruhrberg, Lynda Erskine

**Affiliations:** ^1^ School of Medicine, Medical Sciences and Nutrition, Institute of Medical Sciences, University of Aberdeen, Foresterhill, Aberdeen AB25 2ZD, UK; ^2^ Instituto de Neurociencias de Alicante (Consejo Superior de Investigaciones Científicas-Universidad Miguel Hernández, CSIC-UMH), Campus San Juan, Av. Ramón y Cajal s/n, Alicante 03550, Spain; ^3^ UCL Institute of Ophthalmology, University College London, 11-43 Bath Street, London EC1V 9EL, UK; ^4^ Institute for Pharmacology/Toxicology, Jena University Hospital, Drackendorfer Str. 1, D-07747 Jena, Germany

**Keywords:** Axon guidance, Binocular vision, Growth cone, Meninges, Optic pathway, CXCL12, Mouse

## Abstract

Binocular vision requires the segregation of retinal ganglion cell (RGC) axons extending from the retina into the ipsilateral and contralateral optic tracts. RGC axon segregation occurs at the optic chiasm, which forms at the ventral diencephalon midline. Using expression analyses, retinal explants and genetically modified mice, we demonstrate that CXCL12 (SDF1) is required for axon segregation at the optic chiasm. CXCL12 is expressed by the meninges bordering the optic pathway, and CXCR4 by both ipsilaterally and contralaterally projecting RGCs. CXCL12 or ventral diencephalon meninges potently promoted axon outgrowth from both ipsilaterally and contralaterally projecting RGCs. Further, a higher proportion of axons projected ipsilaterally in mice lacking CXCL12 or its receptor CXCR4 compared with wild-type mice as a result of misrouting of presumptive contralaterally specified RGC axons. Although RGCs also expressed the alternative CXCL12 receptor ACKR3, the optic chiasm developed normally in mice lacking ACKR3. Our data support a model whereby meningeal-derived CXCL12 helps drive axon growth from CXCR4-expressing RGCs towards the diencephalon midline, enabling contralateral axon growth. These findings further our understanding of the molecular and cellular mechanisms controlling optic pathway development.

## Teaser

**Highlighted Article:** This study shows that retinal ganglion cell axon navigation to the contralateral brain hemisphere relies on the chemokine CXCL12 and its receptor CXCR4.

## INTRODUCTION

Visual information is perceived by photoreceptors which transfer their information to visual targets in the brain via the axons from retinal ganglion cells (RGCs). The growth and guidance of RGC axons from the eye during embryonic development is therefore essential for functional vision. RGC axons exit the eyes at the optic disc and extend through the optic nerves to the ventral midline of the diencephalon, where the two nerves meet to form the optic chiasm. In species with binocular vision, such as mice and humans, axons segregate at the optic chiasm to project either to targets on the same (ipsilateral) or opposite (contralateral) side of the brain (Jeffery and Erskine, 2005). The ipsilaterally projecting RGCs originate predominately in the temporal retina and are specified by the transcription factor ZIC2 (Herrera et al., 2003).

Ongoing research seeks to unravel the molecular mechanisms important to segregate ipsilaterally and contralaterally projecting RGC axons at the optic chiasm. Ephrin B2 (EFNB2) is expressed at the diencephalic midline and binds to its receptor EPHB1, which is expressed by ipsilaterally but not contralaterally projecting RGC axons. EPHB1 signalling repels ipsilaterally projecting RGC axons away from the midline and into the ipsilateral optic tract (Williams et al., 2003). Sonic hedgehog released by contralaterally projecting RGC axons also is important for repelling ipsilateral axons away from the midline (Peng et al., 2018). Conversely, vascular endothelial growth factor A (VEGFA) is expressed at the diencephalic midline to promote the growth of contralaterally projecting axons that express the VEGFA receptor NRP1 (Erskine et al., 2017, 2011; Tillo et al., 2015). SEMA6D/PLXNA1/NRCAM interactions also are required for contralateral RGC axon growth (Kuwajima et al., 2012). However, in the absence of any one of these signalling pathways, many RGC axons still segregate correctly at the chiasm. This may reflect functional complementarity and partial redundancy between these molecularly distinct pathways. Alternatively, additional, unidentified guidance cues contribute to axon segregation at the optic chiasm.

As RGC axons extend through the optic pathway, they grow in close proximity to the meninges, the tissue that covers and protects the developing and mature brain (Bovolenta and Mason, 1987; Colello and Guillery, 1992, 1998; Colello and Coleman, 1997). The chemokine CXCL12 (SDF1) is expressed in the pial layer of the meninges and signals through its receptor CXCR4 to regulate multiple aspects of neuronal development, including promoting outgrowth of developing axons (Abe et al., 2015; Arakawa et al., 2003; Borrell and Marín, 2006; Lerner et al., 2010; Mithal et al., 2013; Pritchett et al., 2007; Reiss et al., 2002; Somaa et al., 2015; Zhu et al., 2009, 2002). The alternative CXCL12 receptor ACKR3 (CXCR7) can act as a scavenger receptor for CXCL12 to control the amount of CXCL12 available for binding to CXCR4 (Abe et al., 2014; Sánchez-Alcañiz et al., 2011) or activate downstream signalling pathways in neurons (Wang et al., 2011). CXCL12 signalling through CXCR4 also attenuates the response of RGC axons to inhibitory guidance signals such as Slits, both *in vitro* and *in vivo* (Chalasani et al., 2003b, 2007). However, it is not known whether meninges-derived CXCL12 or its receptors CXCR4 and ACKR3 are essential for RGC axon segregation at the optic chiasm.

Here, we have combined expression and genetic studies with axon explant cultures to examine whether and how CXCL12 and its receptors regulate RGC axon guidance at the optic chiasm. Our findings demonstrate that CXCL12 signalling through CXCR4 is essential for establishing the binocular visual pathways by enabling axon growth towards the chiasm midline.

## RESULTS AND DISCUSSION

### Cxcl12 is expressed by the ventral diencephalon meninges and Cxcr4 by RGCs

To establish the expression pattern of *Cxcl12* and *Cxcr4* relative to the developing optic chiasm, we performed *in situ* hybridisation on vibratome sections of mouse embryo retina and ventral diencephalon. As relevant time points, we chose the period spanning from embryonic day (E)12.5 to E17.5. At E12.5, the first RGCs give rise to contralaterally projecting RGCs and a transient ipsilateral projection (Marcus and Mason, 1995; Soares and Mason, 2015). At E14.5, RGCs within the ventrotemporal crescent of the retina are generated, which give rise to the permanent ipsilateral projection, alongside contralaterally projecting RGCs that originate throughout the retina (Colello and Guillery, 1990; Drager, 1985; Marcucci et al., 2019). At E17.5, only contralaterally projecting RGCs are generated (Godement et al., 1987; Marcucci et al., 2019).

**Fig. 1.**
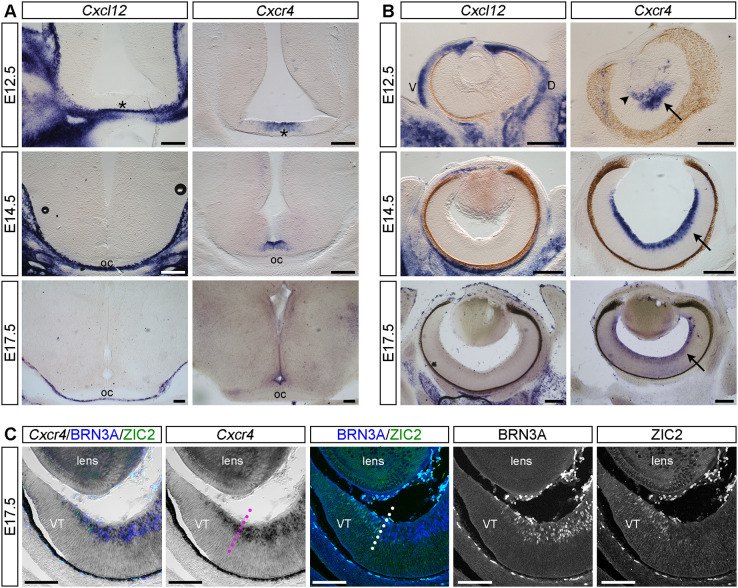
***Cxcl12* is expressed by ventral diencephalon meninges and *Cxcr4* is expressed by RGCs.** (A,B) *In situ* hybridisation for *Cxcl12* and *Cxcr4* on coronal vibratome sections through the ventral diencephalon (A) and retina (B) of E12.5, E14.5 and E17.5 mouse embryos. Asterisks in A indicate the position in the ventral diencephalon where the optic chiasm (oc) will form. In B, arrows indicate expression of *Cxcr4* in the RGC layer of the retina and arrowhead indicates the hyaloid vasculature. (C) Cryosection through the E17.5 peripheral ventrotemporal (VT) retina stained after *Cxcr4 in situ* hybridisation by immunofluorescence with antibodies specific for BRN3A (labels contralaterally projecting RGCs) and ZIC2 (labels ipsilaterally projecting RGCs). Staining is shown as the combined and single channels. Dotted lines indicate the boundary between ZIC2-positive and -negative RGCs. Scale bars: 200 µm (A,B); 100 µm (C). D, dorsal; V, ventral; VT, ventrotemporal.

At all three developmental stages examined, *Cxcl12* was not detected in the diencephalic parenchyma (Fig. 1A) or retina (Fig. 1B). By contrast, *Cxcl12* expression was detected in the meninges adjacent to the developing optic chiasm and tracts (Fig. 1A) and in the mesenchyme around the developing eye and optic nerve (Fig. 1B). At all three developmental stages examined, *Cxcr4* expression was detected at the ventral midline of the diencephalon (Fig. 1A) and in RGCs (Fig. 1B).

To determine whether *Cxcr4* is expressed by both ipsilaterally and contralaterally projecting RGCs, we combined *in situ* hybridisation for *Cxcr4* with immunofluorescent staining for ZIC2, a transcription factor expressed by ipsilaterally but not contralaterally projecting RGCs (Herrera et al., 2003), and for BRN3A (POU4F1), a transcription factor expressed by contralaterally projecting RGCs (Quina et al., 2005). Staining was performed in cryosections through the ventrotemporal region of E17.5 mouse retinas (Fig. 1C). As expected, ZIC2 localised to RGCs within the peripheral ventrotemporal retina, whereas BRN3A-positive RGCs were broadly distributed throughout the remainder of the retina. *Cxcr4* was expressed in both ZIC2-positive and BRN3A-positive RGCs (Fig. 1C).

Taken together, *Cxcl12* is expressed by the ventral diencephalon meninges adjacent to the developing optic chiasm and tracts*,* whereas its receptor, *Cxcr4,* is expressed by both ipsilaterally and contralaterally projecting RGCs.

### CXCL12 and CXCR4 are required for contralateral growth at the optic chiasm

**Fig. 2.**
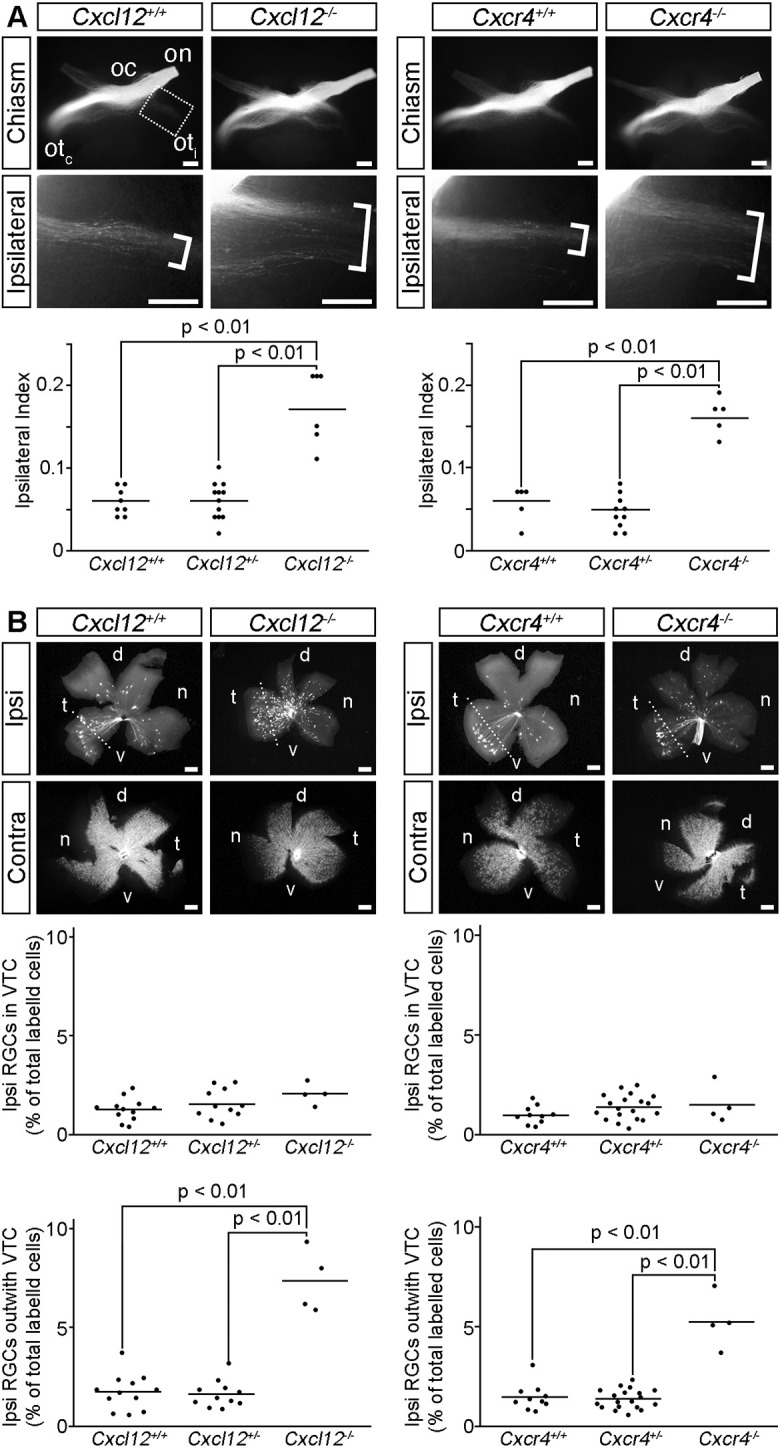
**CXCL12 and CXCR4 are required for contralateral RGC axon growth at the optic chiasm.** (A) Ventral views of RGC axons labelled from one eye in E14.5 *Cxcl12* and *Cxcr4* wild-type and mutant littermates captured using a stereo microscope. The optic nerve (on), optic chiasm (oc), contralateral optic tract (ot_c_) and ipsilateral optic tract (ot_i_) are indicated. Boxed region is shown at higher magnification in the lower panels; brackets indicate the width of the ipsilateral projection. Number analysed of each genotype was: *Cxcl12^+/+^ n*=8, *Cxcl12^+/−^ n*=12, *Cxcl12^−/−^ n*=6; *Cxcr4^+/+^ n*=5, *Cxcr4^+/−^ n*=10, *Cxcr4^−/−^ n*=5. (B) Ipsilateral (Ipsi) and contralateral (Contra) flatmounted retinas from retrogradely labelled E15.5 *Cxcl12* and *Cxcr4* wild-type and mutant littermates. Dotted line demarcates the ventrotemporal crescent (VTC). d, dorsal; n, nasal; t, temporal; v, ventral. Number analysed of each genotype was: *Cxcl12^+/+^ n*=12, *Cxcl12^+/−^ n*=11, *Cxcl12^−/−^ n*=4; *Cxcr4^+/+^ n*=10, *Cxcr4^+/−^ n*=19, *Cxcr4^−/−^ n*=4. Graphs display quantification of the Ipsilateral Index (A) and proportion of ipsilaterally projecting RGCs relative to the total number of labelled cells in both eyes (B), with each dot representing the value for one embryo and horizontal bars the mean values. Statistical analyses were performed using one-way ANOVA with Tukey post-hoc comparison. Scale bars: 200 µm.

To determine whether CXCL12 and CXCR4 regulate RGC axon guidance at the optic chiasm, we used the lipophilic dye DiI to anterogradely and retrogradely label RGC axons in *Cxcl12* and *Cxcr4* mutants and their wild-type littermates (Fig. 2). As expected (Erskine et al., 2017, 2011), anterograde labelling of RGC axons from one eye of E14.5 wild-type embryos showed that RGC axons extended along the optic nerve, and the majority crossed at the optic chiasm to project into the contralateral optic tract (Fig. 2A). Both *Cxcl12^−/−^* and *Cxcr4^−/−^* embryos displayed a substantial increase in the proportion of axons projecting ipsilaterally (Fig. 2A). Accordingly, the ipsilateral index, defined as the ratio of the fluorescent intensity in a defined region of the ipsilateral optic tract relative to the sum of the fluorescent intensity in comparable regions of the ipsilateral and contralateral optic tracts (Erskine et al., 2011), was increased significantly in both *Cxcl12* and *Cxcr4* mutants compared with heterozygous and wild-type littermates (Fig. 2A). The ipsilateral optic tracts of *Cxcl12* and *Cxcr4* mutants also occupied a broader domain than in wild-type embryos (Fig. 2A; brackets). This abnormal tract morphology may reflect the alignment of misrouted contralaterally fated RGC axons with contralateral (rather than ipsilateral) axons from the other eye.

Unilateral retrograde DiI labelling of RGCs from the dorsal thalamus to the ipsilateral and contralateral retinas showed that ipsilaterally projecting RGCs were located predominately in the ventrotemporal crescent of the wild-type retina (Fig. 2B), as expected (Drager, 1985; Erskine et al., 2017; Marcucci et al., 2019; Tillo et al., 2015). Consistent with normal specification and guidance of ipsilaterally specified RGCs, the relative number of ipsilaterally projecting RGCs within the ventrotemporal crescent was not altered in *Cxcl12* or *Cxcr4* mutants (Fig. 2B). In contrast, the proportion of ipsilaterally projecting RGCs originating in regions of the retina that normally give rise to contralaterally projecting RGCs was increased significantly in *Cxcl12* and *Cxcr4* mutants compared with wild-type and heterozygous littermates (Fig. 2B).

Together, these findings suggest that presumptive contralaterally projecting RGCs are misrouted into the ipsilateral optic tract when CXCL12 or CXCR4 are absent.

### CXCL12 loss does not impair RGC density or optic chiasm midline morphology

Although *Cxcl12* is not obviously expressed in the E14.5 retina (Fig. 1B), we nevertheless sought to exclude that optic chiasm defects in the absence of CXCL12 were due to obvious changes within the developing retina or disorganisation of the optic chiasm midline where *Cxcr4* is expressed (Fig. 1A,B). We found that the number of phosphohistone-H3-positive mitotic cells was similar in E14.5 *Cxcl12* mutant retinas compared with their heterozygous and wild-type littermates (Fig. S1A). Furthermore, the density of BRN3A-positive RGCs was not significantly different in *Cxcl12* mutants compared with heterozygous and wild-type littermates (Fig. S1B). Staining sections through the optic chiasm midline of E14.5 *Cxcl12* mutant and wild-type littermates with the radial glial marker RC2 (NES; Marcus et al., 1995; Marcus and Mason, 1995) showed that the overall organisation of the midline appeared grossly normal, but a few fibres appeared to be less organised and deviated from their normal radial trajectory (Fig. S2; blue arrows). This finding may suggest a role for meningeal-derived CXCL12 in radial glia anchorage within the ventral diencephalon, in analogy to the spinal cord, where CXCL12 promotes radial glia process arborisation and endfeet formation (Mithal et al., 2013). We consider it unlikely that these subtle changes in radial glial cell organisation are sufficient to cause RGC axon misrouting.

Together, these findings suggest that changes in retina or ventral diencephalon glia development in *Cxcl12* mutants do not underlie the altered RGC axon segregation at the optic chiasm.

### ACKR3 is dispensable for axon guidance at the optic chiasm

**Fig. 3.**
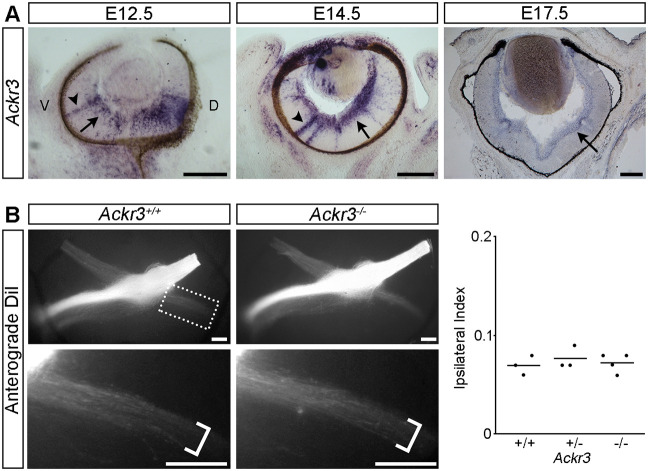
**ACKR3 is dispensable for RGC axon guidance at the optic chiasm.** (A) *In situ* hybridisation of coronal sections from E12.5, E14.5 and E17.5 wild-type mouse eyes with a probe specific for *Ackr3*. Arrows indicate expression in the RGC layer, arrowheads indicate expression in radially oriented cells. D, dorsal; V, ventral. (B) Ventral views of the optic pathway anterogradely labelled with DiI in E14.5 *Ackr3* wild-type and mutant embryos. Boxed region is shown at higher magnification in the lower panels, brackets indicate the width of the ipsilateral projection. Number analysed of each genotype was: *Ackr3^+/+^ n*=3, *Ackr3^−/+^ n*=3, *Ackr3^−/−^ n*=4. Graph displays quantification of the Ipsilateral Index with each dot representing the value for one embryo and horizontal bars the mean values. Scale bars: 200 µm.

*In situ* hybridisation demonstrated that *Ackr3* was expressed by RGCs from E12.5 to E17.5 (Fig. 3A, arrows). At E12.5 and E14.5, expression was also detected in radial cells extending throughout the depth of the retina (Fig. 3A, arrowheads). These cells may be nascent RGCs that have not yet retracted their apical process and translocated into the RGC layer (Hinds and Hinds, 1974; McLoon and Barnes, 1989), thus raising the possibility that *Ackr3* is expressed by RGCs shortly after their terminal differentiation. Nevertheless, anterograde DiI labelling of RGCs from one eye of E14.5 *Ackr3* wild-type, heterozygous and mutant littermates revealed no obvious differences in the size or organisation of the contralateral or ipsilateral optic tracts (Fig. 3B). The ipsilateral index was also similar in *Ackr3* mutants compared with their heterozygous and wild-type littermates (Fig. 3B). Accordingly, ACKR3 is expressed by RGCs throughout the period when the optic pathway forms but is dispensable for RGC axon guidance at the optic chiasm.

### CXCL12 and ventral diencephalon meninges promote RGC axon outgrowth *in vitro*

**Fig. 4.**
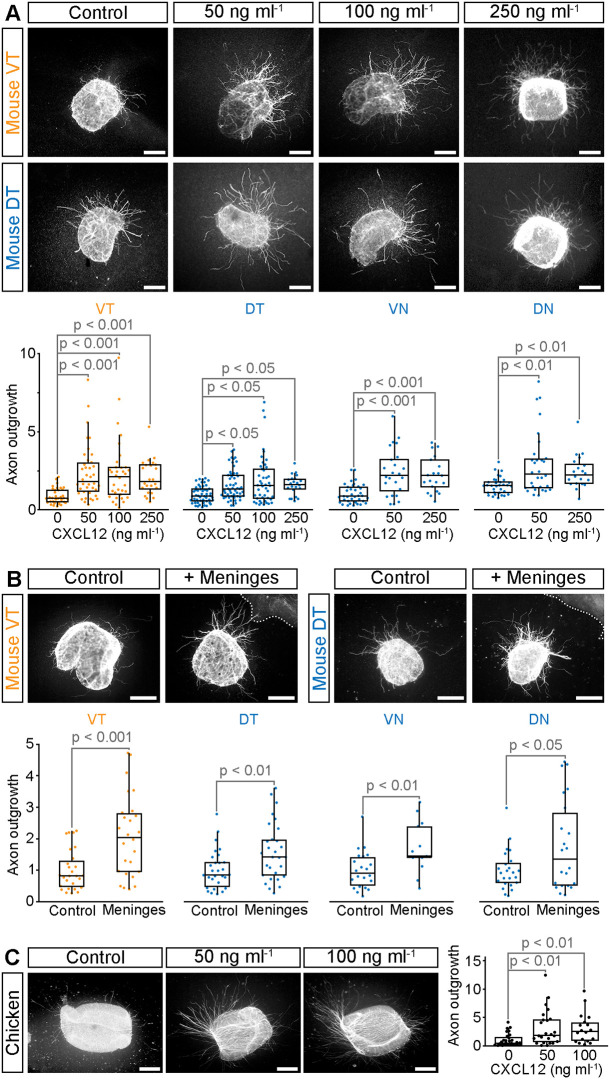
**CXCL12 and ventral diencephalon meninges promote RGC axon outgrowth.** (A-C) Representative examples and quantification of E14.5 mouse (A,B) or E6 chicken (C) retinal explants cultured in the presence and absence of CXCL12 (A,C) or ventral diencephalon meninges (B). Mouse explants from peripheral ventrotemporal (VT) retina contain predominately ipsilaterally projecting RGCs; peripheral dorsotemporal (DT), ventronasal (VN) and dorsonasal (DN) explants contain contralaterally projecting RGCs. All RGCs in chicken project contralaterally. Dotted lines in B indicate the edge of the meningeal tissue. Quantitative data are shown as boxplots with the median values (middle bars) and first to third interquartile ranges (boxes); whiskers indicate 1.5× the interquartile ranges; each dot represents the value from one explant. A minimum of 22 explants from three independent experiments (A), 13 explants from three independent experiments (B) or 18 explants from four independent experiments (C) were analysed per condition. Statistical analyses were performed using Kruskal–Wallis rank sum test with TUKEY-Kramer post-hoc comparison. Scale bars: 200 µm.

To investigate whether CXCL12 is growth-promoting for RGC axons, we cultured explants from E14.5 wild-type mouse retinas in collagen gels in the presence or absence of CXCL12. Explants were taken from the peripheral ventrotemporal crescent, which gives rise to ipsilaterally projecting RGCs or peripheral dorsotemporal, ventronasal and dorsonasal retina, all sources of contralaterally projecting RGCs. Culturing mouse retinal explants with CXCL12 significantly increased axon outgrowth from RGCs from all four retinal quadrants at the concentrations tested (50-250 ng ml^−1^; Fig. 4A). Outgrowth of RGC axons from all four retinal quadrants also was increased significantly by culturing the explants in collagen at a short distance (100-400 µm) from ventral diencephalon meninges (Fig. 4B), which express CXCL12 (Fig. 1A). Although previous work suggested that CXCL12 does not collapse or attract chicken RGC growth cones (Chalasani et al., 2003b), we found that CXCL12 instead had a growth promoting effect on chicken RGC axons (Fig. 4C), as observed for mouse. These findings demonstrate that CXCL12 is a potent promoter of RGC axon outgrowth, and that this activity is conserved between mouse and chicken.

The finding that RGC axon outgrowth from all four retinal quadrants is stimulated by CXCL12 *in vitro* is consistent with *Cxcr4* expression by both ipsilaterally and contralaterally projecting RGC axons (Fig. 1C). VEGFA and SEMA6D are also required for contralateral RGC axon growth at the optic chiasm. However, they differ from CXCL12 by selectively promoting contralateral RGC axon growth *in vitro*, consistent with their receptors being expressed specifically by contralaterally projecting RGCs (Erskine et al., 2011; Fernández-Nogales et al., 2022; Kuwajima et al., 2012). The selective requirement of CXCL12 by RGC axons for contralateral growth *in vivo* (Fig. 2) may therefore be explained by a greater dependence on growth promoting cues for axon growth across the midline as opposed to deflection into the ipsilateral tract.

In addition to a direct effect on RGC axon outgrowth, CXCL12 can also indirectly modulate axon outgrowth through dampening the response of axons to inhibitory guidance signals such as Slits (Chalasani et al., 2003b, 2007), which are expressed in the ventral diencephalon and inhibit outgrowth of both ipsilaterally and contralaterally projecting RGC axons (Erskine et al., 2000; Niclou et al., 2000; Plump et al., 2002; Rafipay et al., 2021). Consistent with this idea, cultured ventral diencephalon cells and tissues with the meninges removed inhibit outgrowth of both ipsilaterally and contralaterally projecting RGC axons (Wang et al., 1995, 1996). CXCL12 may therefore play a dual role in facilitating contralateral growth at the optic chiasm: directly promoting RGC axon outgrowth and indirectly decreasing the effectiveness of growth-inhibitory signals such as Slits, which emerge from the ventral diencephalon.

Taking our findings as a whole, we propose the following working model for the role of CXCL12 in optic chiasm development: CXCL12 is produced by the pial layer of the meninges to promote the growth of both ipsilaterally and contralaterally projecting RGC axons as they exit the optic nerve and extend into the ventral diencephalon. Concurrently, CXCL12 dampens the response of RGC axons to inhibitory signals within the ventral diencephalon, thereby also helping facilitate growth towards the chiasm midline. In the absence of CXCL12 (or CXCR4) the ability of RGC axons to grow through the complex diencephalic environment is impaired, resulting in some contralaterally projecting axons being unable to reach the ventral midline and aberrantly entering the ipsilateral optic tract. In contrast, because ipsilaterally specified RGC axons are normally repelled away from the midline (Peng et al., 2018; Williams et al., 2003), reduced fidelity of growth toward the ventral diencephalon midline does not impact on the routing of these axons.

### Conclusion

By combining genetic and *in vitro* analyses, we have shown here that CXCL12 signalling through CXCR4 promotes the contralateral growth of RGC axons at the optic chiasm, essential for the correct establishment of the binocular visual pathways. As CXCL12 also promotes the growth of adult RGC axons and, therefore, regeneration following optic nerve injury (Xie et al., 2022), our findings support the concept that shared pathways facilitate RGC axon growth for optic pathway development and regeneration (Varadarajan et al., 2022).

## MATERIALS AND METHODS

### Animals and tissue preparation

Animal procedures were performed in accordance with institutional Animal Welfare and Ethical Review Board and UK Home Office guidelines. The following mouse strains were used: wild-type C57Bl/6J mice from an in-house breeding colony, as well as *Cxcl12^+/−^* (Ara et al., 2003), *Cxcr4^+/−^* (Ivins et al., 2015) and *Ackr3^+/−^* (Haege et al., 2012) on a C57Bl/6J background. In timed matings, noon on the day a vaginal plug was found was defined as E0.5. Embryos were either fixed overnight with 4% formaldehyde in PBS for sectioning or the retinas were dissected and used without fixation for tissue culture experiments. Fertilised Dekalb White chicken eggs were obtained from Medeggs, Norfolk, UK. Eggs were incubated on their side in a 37°C incubator to E6 and the retinas dissected for culture experiments.

### *In situ* hybridisation

Antisense probes were generated using DIG-labelled nucleotides (Merck Life Science) for *Cxcl12* (Stumm et al., 2002), *Cxcr4* (Chalasani et al., 2003a) and *Ackr3* (Sánchez-Alcañiz et al., 2011). *In situ* hybridisation on vibratome sections was performed as previously described (Erskine et al., 2000; Thompson et al., 2006) using 100 µm coronal sections of E12.5-E17.5 fixed mouse heads embedded in 3% agarose. *In situ* hybridisation on cryostat sections was performed on fixed embryo heads cryoprotected in 30% sucrose in PBS, embedded in a 1:1 mixture of OCT (Thermo Fisher Scientific) and PBS and frozen in a bath of isopentane in dry ice for coronal sectioning at 25 µm. Images were captured using a Nikon SMZ1500 microscope with a Nikon DS-Fi1c digital camera.

### Immunostaining

Immunostaining was performed on cryosections prepared as described above or on retinas dissected from fixed embryos, with a cut marking the nasal side to enable orientation. The tissue was washed with PBS, blocked with PBS containing 10% goat serum and 0.2% Triton X-100 and incubated at 4°C overnight for cryosections or 2 days for retinas with the following antibodies in blocking solution: monoclonal antibody RC2 (1:5; Developmental Studies Hybridoma Bank, AB_531887) or mouse anti-BRN3A (1:100; Merck Life Sciences, MAB1585) in combination with rabbit anti-mouse ZIC2 (Murillo et al., 2015) or rabbit anti-phosphohistone-H3 (1:100; Merck Life Sciences, 06-570). The tissue was washed with PBS and incubated in the appropriate secondary antibody: Cy3-conjugated goat anti-mouse IgM (1:1500; Jackson ImmunoResearch, RC2), Alexa Fluor-488 goat anti-mouse IgG (1:500; Thermo Fisher Scientific, BRN3A) or Cy3-conjugated goat anti-rabbit IgG (1:1500; Jackson ImmunoResearch, ZIC2 or phosphohistone-H3). The tissue was washed with PBS and mounted in Vectashield Antifade Mounting Medium with DAPI (Vector Laboratories). Images were captured using a Zeiss LSM710 or LSM880 confocal microscope or a Zeiss Axiophot Microscope with a Nikon DXM1200 camera.

For quantification of BRN3A-labelled cells, a comparable region from all four quadrants of each retina was photographed at 50× using a Zeiss Axiophot microscope, the number of labelled cells per image counted manually and the cell density calculated. For quantification of phosphohistone-H3-positive cells, images of the entire retina were captured at 10×, merged using Adobe Photoshop and the number of labelled cells counted in ImageJ (https://imagej.nih.gov/ij/index.html) using the Analyse Particles function on thresholded images.

### Anterograde and retrograde DiI labelling of RGC axons

For anterograde labelling of all RGC axons from one eye, a small crystal of DiIC_18_ (Thermo Fisher Scientific) was placed over the optic disc of one eye in formaldehyde-fixed embryos in PBS. After 3-4 days at 37°C, the diencephalon was dissected, photographed ventral side up and the relative size of the ipsilateral projection (ipsilateral index) quantified as previously described (Erskine et al., 2011). For retrograde DiI labelling of RGCs, the cortex was removed from one side of the brain and DiI crystals placed in a row over the dorsal thalamus. After incubation at room temperature in PBS for 8-12 weeks, the ipsilateral and contralateral retinas were dissected and flatmounted in VectaShield Antifade Mounting Medium (Erskine et al., 2017). The total number of labelled RGCs in each retina was counted manually and the percentage of ipsilaterally projecting RGCs to the total amount of labelled RGCs in both the ipsilateral and contralateral retinas calculated. All tissues were photographed using a Nikon SMZ1500 microscope with a DXM1200 camera.

### Retinal explant cultures

Peripheral explants from each quadrant of E14.5 wild-type mouse retinas or from E6 chicken retinas were placed in a 1:1 mixture of bovine dermis collagen and rat tail collagen (Corning) as previously described (Erskine et al., 2011, 2000). The explants were treated with 0-250 ng ml^−1^ recombinant mouse CXCL12 (Bio-Techne, 460-SD-010), 0-100 ng ml^−1^ recombinant chicken CXCL12 (Cambridge Bioscience, RP1354CT) or positioned 100-400 µm from pieces of ventral diencephalon meninges. After 24 h, the cultures were fixed with 4% formaldehyde in PBS, blocked with PBS containing 10% goat serum and 0.2% Triton X-100 and stained with neuron-specific anti-β-tubulin III (1:500 in blocking solution; Merck Life Sciences, T8660) followed by Cy3-conjugated goat anti-mouse IgG (1:2000 in PBS containing 1% goat serum). The gels were mounted in Vectashield Antifade Mounting Medium and photographed using a Zeiss Axiophot or Nikon DXM1200 microscope with a DXM1200 camera. The area of axon outgrowth was analysed using the plug-in Neurite-J (Torres-Espín et al., 2014). For each experiment, outgrowth was normalised to the mean outgrowth in the relevant control condition.

### Data presentation and statistical analyses

Graphs were plotted using PlotsofData (https://huygens.science.uva.nl/PlotsOfData/) using quasirandom data offset and displaying mean values or boxplots. Normality of datasets was assessed using the Shapiro-Wilks Normality Test. For normally distributed data, statistical analyses were performed using one-way ANOVA with TUKEY post-hoc comparison. For data that were not normally distributed, analyses were performed using the Mann–Whitney *U*-test or Kruskal–Wallis rank sum test with TUKEY-Kramer post-hoc comparison. All analyses were conducted unaware of genotypes or treatment.

## Supplementary Material

10.1242/develop.202446_sup1 Supplementary information

## References

[DEV202446C1] Abe, P., Mueller, W., Schütz, D., Mackay, F., Thelen, M., Zhang, P. and Stumm, R. (2014). CXCR7 prevents excessive CXCL12-mediated downregulation of CXCR4 in migrating cortical interneurons. *Development* 141, 1857-1863. 10.1242/dev.104224 24718993

[DEV202446C2] Abe, P., Molnár, Z., Tzeng, Y.-S., Lai, D.-M., Arnold, S. J. and Stumm, R. (2015). Intermediate progenitors facilitate intracortical progression of thalamocortical axons and interneurons through CXCL12 chemokine signaling. *J. Neurosci.* 35, 13053-13063. 10.1523/JNEUROSCI.1488-15.2015 26400936 PMC6605439

[DEV202446C3] Ara, T., Nakamura, Y., Egawa, T., Sugiyama, T., Abe, K., Kishimoto, T., Matsui, Y. and Nagasawa, T. (2003). Impaired colonization of the gonads by primordial germ cells in mice lacking a chemokine, stromal cell-derived factor-1 (SDF-1). *Proc. Natl. Acad. Sci. USA* 100, 5319-5323. 10.1073/pnas.0730719100 12684531 PMC154343

[DEV202446C4] Arakawa, Y., Bito, H., Furuyashiki, T., Tsuji, T., Takemoto-Kimura, S., Kimura, K., Nozaki, K., Hashimoto, N. and Narumiya, S. (2003). Control of axon elongation via an SDF-1α/Rho/mDia pathway in cultured cerebellar granule neurons. *J. Cell Biol.* 161, 381-391. 10.1083/jcb.200210149 12707308 PMC2172896

[DEV202446C5] Borrell, V. and Marín, O. (2006). Meninges control tangential migration of hem-derived Cajal-Retzius cells via CXCL12/CXCR4 signaling. *Nat. Neurosci.* 9, 1284-1293. 10.1038/nn1764 16964252

[DEV202446C6] Bovolenta, P. and Mason, C. (1987). Growth cone morphology varies with position in the developing mouse visual pathway from retina to first targets. *J. Neurosci.* 7, 1447-1460. 10.1523/JNEUROSCI.07-05-01447.1987 3572487 PMC6568811

[DEV202446C7] Chalasani, S. H., Baribaud, F., Coughlan, C. M., Sunshine, M. J., Lee, V. M. Y., Doms, R. W., Littman, D. R. and Raper, J. A. (2003a). The chemokine stromal cell-derived factor-1 promotes the survival of embryonic retinal ganglion cells. *J. Neurosci.* 23, 4601-4612. 10.1523/JNEUROSCI.23-11-04601.2003 12805300 PMC6740796

[DEV202446C8] Chalasani, S. H., Sabelko, K. A., Sunshine, M. J., Littman, D. R. and Raper, J. A. (2003b). A chemokine, SDF-1, reduces the effectiveness of multiple axonal repellents and is required for normal axon pathfinding. *J. Neurosci.* 23, 1360-1371. 10.1523/JNEUROSCI.23-04-01360.2003 12598624 PMC6742262

[DEV202446C9] Chalasani, S. H., Sabol, A., Xu, H., Gyda, M. A., Rasband, K., Granato, M., Chien, C.-B. and Raper, J. A. (2007). Stromal cell-derived factor-1 antagonizes slit/robo signaling in vivo. *J. Neurosci.* 27, 973-980. 10.1523/JNEUROSCI.4132-06.2007 17267551 PMC6673187

[DEV202446C10] Colello, S. J. and Coleman, L.-A. (1997). Changing course of growing axons in the optic chiasm of the mouse. *J. Comp. Neurol.* 379, 495-514. 10.1002/(SICI)1096-9861(19970324)379:4<495::AID-CNE3>3.0.CO;2-Y 9067839

[DEV202446C11] Colello, R. J. and Guillery, R. W. (1990). The early development of retinal ganglion cells with uncrossed axons in the mouse: retinal position and axonal course. *Development* 108, 515-523. 10.1242/dev.108.3.515 2340812

[DEV202446C12] Colello, R. J. and Guillery, R. W. (1992). Observations on the early development of the optic nerve and tract of the mouse. *J. Comp. Neurol.* 317, 357-378. 10.1002/cne.903170404 1578002

[DEV202446C13] Colello, S. J. and Guillery, R. W. (1998). The changing pattern of fibre bundles that pass through the optic chiasm of mice. *Eur. J. Neurosci.* 10, 3653-3663. 10.1046/j.1460-9568.1998.00416.x 9875344

[DEV202446C14] Drager, U. C. (1985). Birth dates of retinal ganglion cells giving rise to the crossed and uncrossed optic projections in the mouse. *Proc. R. Soc. Lond. B Biol. Sci.* 224, 57-77. 10.1098/rspb.1985.0021 2581263

[DEV202446C15] Erskine, L., Williams, S. E., Brose, K., Kidd, T., Rachel, R. A., Goodman, C. S., Tessier-Lavigne, M. and Mason, C. A. (2000). Retinal ganglion cell axon guidance in the mouse optic chiasm: expression and function of robos and slits. *J. Neurosci.* 20, 4975-4982. 10.1523/JNEUROSCI.20-13-04975.2000 10864955 PMC6772295

[DEV202446C16] Erskine, L., Reijntjes, S., Pratt, T., Denti, L., Schwarz, Q., Vieira, J. M., Alakakone, B., Shewan, D. and Ruhrberg, C. (2011). VEGF signaling through neuropilin 1 guides commissural axon crossing at the optic chiasm. *Neuron* 70, 951-965. 10.1016/j.neuron.2011.02.052 21658587 PMC3114076

[DEV202446C17] Erskine, L., François, U., Denti, L., Joyce, A., Tillo, M., Bruce, F., Vargesson, N. and Ruhrberg, C. (2017). VEGF-A and neuropilin 1 (NRP1) shape axon projections in the developing CNS via dual roles in neurons and blood vessels. *Development* 144, 2504-2516. 10.1242/dev.151621 28676569 PMC5536872

[DEV202446C18] Fernández-Nogales, M., López-Cascales, M. T., Murcia-Belmonte, V., Escalante, A., Fernández-Albert, J., Muñoz-Viana, R., Barco, A. and Herrera, E. (2022). Multiomic analysis of neurons with divergent projection patterns identifies novel regulators of axon pathfinding. *Adv. Sci. (Weinh)* 9, e2200615. 10.1002/advs.202200615 35988153 PMC9561852

[DEV202446C19] Godement, P., Vanselow, J., Thanos, S. and Bonhoeffer, F. (1987). A study in developing visual systems with a new method of staining neurones and their processes in fixed tissue. *Development* 101, 697-713. 10.1242/dev.101.4.697 2460302

[DEV202446C20] Haege, S., Einer, C., Thiele, S., Mueller, W., Nietzsche, S., Lupp, A., Mackay, F., Schulz, S. and Stumm, R. (2012). CXC chemokine receptor 7 (CXCR7) regulates CXCR4 protein expression and capillary tuft development in mouse kidney. *PLoS ONE* 7, e42814. 10.1371/journal.pone.0042814 22880115 PMC3412803

[DEV202446C21] Herrera, E., Brown, L., Aruga, J., Rachel, R. A., Dolen, G., Mikoshiba, K., Brown, S. and Mason, C. A. (2003). Zic2 patterns binocular vision by specifying the uncrossed retinal projection. *Cell* 114, 545-557. 10.1016/S0092-8674(03)00684-6 13678579

[DEV202446C22] Hinds, J. W. and Hinds, P. L. (1974). Early ganglion cell differentiation in the mouse retina: an electron microscopic analysis utilizing serial sections. *Dev. Biol.* 37, 381-416. 10.1016/0012-1606(74)90156-0 4826283

[DEV202446C23] Ivins, S., Chappell, J., Vernay, B., Suntharalingham, J., Martineau, A., Mohun, T. J. and Scambler, P. J. (2015). The CXCL12/CXCR4 axis plays a critical role in coronary artery development. *Dev. Cell* 33, 455-468. 10.1016/j.devcel.2015.03.026 26017770 PMC4448146

[DEV202446C24] Jeffery, G. and Erskine, L. (2005). Variations in the architecture and development of the vertebrate optic chiasm. *Prog. Retin. Eye Res.* 24, 721-753. 10.1016/j.preteyeres.2005.04.005 16027026

[DEV202446C25] Kuwajima, T., Yoshida, Y., Takegahara, N., Petros, T. J., Kumanogoh, A., Jessell, T. M., Sakurai, T. and Mason, C. (2012). Optic chiasm presentation of Semaphorin6D in the context of Plexin-A1 and Nr-CAM promotes retinal axon midline crossing. *Neuron* 74, 676-690. 10.1016/j.neuron.2012.03.025 22632726 PMC3361695

[DEV202446C26] Lerner, O., Davenport, D., Patel, P., Psatha, M., Lieberam, I. and Guthrie, S. (2010). Stromal cell-derived factor-1 and hepatocyte growth factor guide axon projections to the extraocular muscles. *Dev. Neurobiol.* 70, 549-564. 10.1002/dneu.20796 20506246

[DEV202446C27] Marcucci, F., Soares, C. A. and Mason, C. (2019). Distinct timing of neurogenesis of ipsilateral and contralateral retinal ganglion cells. *J. Comp. Neurol.* 527, 212-224. 10.1002/cne.24467 29761490 PMC6237670

[DEV202446C28] Marcus, R. C. and Mason, C. A. (1995). The first retinal axon growth in the mouse optic chiasm: axon patterning and the cellular environment. *J. Neurosci.* 15, 6389-6402. 10.1523/JNEUROSCI.15-10-06389.1995 7472403 PMC6577988

[DEV202446C29] Marcus, R. C., Blazeski, R., Godement, P. and Mason, C. A. (1995). Retinal axon divergence in the optic chiasm: uncrossed axons diverge from crossed axons within a midline glial specialization. *J. Neurosci.* 15, 3716-3729. 10.1523/JNEUROSCI.15-05-03716.1995 7751940 PMC6578213

[DEV202446C30] McLoon, S. C. and Barnes, R. B. (1989). Early differentiation of retinal ganglion cells: an axonal protein expressed by premigratory and migrating retinal ganglion cells. *J. Neurosci.* 9, 1424-1432. 10.1523/JNEUROSCI.09-04-01424.1989 2703885 PMC6569860

[DEV202446C31] Mithal, D. S., Ren, D. and Miller, R. J. (2013). CXCR4 signaling regulates radial glial morphology and cell fate during embryonic spinal cord development. *Glia* 61, 1288-1305. 10.1002/glia.22515 23828719

[DEV202446C32] Murillo, B., Ruiz-Reig, N., Herrera, M., Fairén, A. and Herrera, E. (2015). Zic2 controls the migration of specific neuronal populations in the developing forebrain. *J. Neurosci.* 35, 11266-11280. 10.1523/JNEUROSCI.0779-15.2015 26269635 PMC6605128

[DEV202446C33] Niclou, S. P., Jia, L. and Raper, J. A. (2000). Slit2 is a repellent for retinal ganglion cell axons. *J. Neurosci.* 20, 4962-4974. 10.1523/JNEUROSCI.20-13-04962.2000 10864954 PMC6772294

[DEV202446C34] Peng, J., Fabre, P. J., Dolique, T., Swikert, S. M., Kermasson, L., Shimogori, T. and Charron, F. (2018). Sonic Hedgehog is a remotely produced cue that controls axon guidance trans-axonally at a midline choice point. *Neuron* 97, 326-340.e324. 10.1016/j.neuron.2017.12.028 29346753

[DEV202446C35] Plump, A. S., Erskine, L., Sabatier, C., Brose, K., Epstein, C. J., Goodman, C. S., Mason, C. A. and Tessier-Lavigne, M. (2002). Slit1 and Slit2 cooperate to prevent premature midline crossing of retinal axons in the mouse visual system. *Neuron* 33, 219-232. 10.1016/S0896-6273(01)00586-4 11804570

[DEV202446C36] Pritchett, J., Wright, C., Zeef, L. and Nadarajah, B. (2007). Stromal derived factor-1 exerts differential regulation on distinct cortical cell populations in vitro. *BMC Dev. Biol.* 7, 31. 10.1186/1471-213X-7-31 17425785 PMC1854892

[DEV202446C37] Quina, L. A., Pak, W., Lanier, J., Banwait, P., Gratwick, K., Liu, Y., Velasquez, T., O'Leary, D. D. M., Goulding, M. and Turner, E. E. (2005). Brn3a-expressing retinal ganglion cells project specifically to thalamocortical and collicular visual pathways. *J. Neurosci.* 25, 11595-11604. 10.1523/JNEUROSCI.2837-05.2005 16354917 PMC6726022

[DEV202446C38] Rafipay, A., Dun, X. P., Parkinson, D. B., Erskine, L. and Vargesson, N. (2021). Knockdown of slit signaling during limb development leads to a reduction in humerus length. *Dev. Dyn.* 250, 1340-1357. 10.1002/dvdy.284 33347679

[DEV202446C39] Reiss, K., Mentlein, R., Sievers, J. and Hartmann, D. (2002). Stromal cell-derived factor 1 is secreted by meningeal cells and acts as chemotactic factor on neuronal stem cells of the cerebellar external granular layer. *Neuroscience* 115, 295-305. 10.1016/S0306-4522(02)00307-X 12401342

[DEV202446C40] Sánchez-Alcañiz, J. A., Haege, S., Mueller, W., Pla, R., Mackay, F., Schulz, S., López-Bendito, G., Stumm, R. and Marín, O. (2011). Cxcr7 controls neuronal migration by regulating chemokine responsiveness. *Neuron* 69, 77-90. 10.1016/j.neuron.2010.12.006 21220100

[DEV202446C41] Soares, C. A. and Mason, C. A. (2015). Transient ipsilateral retinal ganglion cell projections to the brain: Extent, targeting, and disappearance. *Dev. Neurobiol.* 75, 1385-1401. 10.1002/dneu.22291 25788284 PMC4575261

[DEV202446C42] Somaa, F. A., Bye, C. R., Thompson, L. H. and Parish, C. L. (2015). Meningeal cells influence midbrain development and the engraftment of dopamine progenitors in Parkinsonian mice. *Exp. Neurol.* 267, 30-41. 10.1016/j.expneurol.2015.02.017 25708989

[DEV202446C43] Stumm, R. K., Rummel, J., Junker, V., Culmsee, C., Pfeiffer, M., Krieglstein, J., Höllt, V. and Schulz, S. (2002). A dual role for the SDF-1/CXCR4 chemokine receptor system in adult brain: isoform-selective regulation of SDF-1 expression modulates CXCR4-dependent neuronal plasticity and cerebral leukocyte recruitment after focal ischemia. *J. Neurosci.* 22, 5865-5878. 10.1523/JNEUROSCI.22-14-05865.2002 12122049 PMC6757949

[DEV202446C44] Thompson, H., Barker, D., Camand, O. and Erskine, L. (2006). Slits contribute to the guidance of retinal ganglion cell axons in the mammalian optic tract. *Dev. Biol.* 296, 476-484. 10.1016/j.ydbio.2006.06.017 16828733

[DEV202446C45] Tillo, M., Erskine, L., Cariboni, A., Fantin, A., Joyce, A., Denti, L. and Ruhrberg, C. (2015). VEGF189 binds NRP1 and is sufficient for VEGF/NRP1-dependent neuronal patterning in the developing brain. *Development* 142, 314-319. 10.1242/dev.115998 25519242 PMC4302834

[DEV202446C46] Torres-Espín, A., Santos, D., González-Pérez, F., Del Valle, J. and Navarro, X. (2014). Neurite-J: an image-J plug-in for axonal growth analysis in organotypic cultures. *J. Neurosci. Methods* 236, 26-39. 10.1016/j.jneumeth.2014.08.005 25124852

[DEV202446C47] Varadarajan, S. G., Hunyara, J. L., Hamilton, N. R., Kolodkin, A. L. and Huberman, A. D. (2022). Central nervous system regeneration. *Cell* 185, 77-94. 10.1016/j.cell.2021.10.029 34995518 PMC10896592

[DEV202446C48] Wang, L.-C., Dani, J., Godement, P., Marcus, R. C. and Mason, C. A. (1995). Crossed and uncrossed retinal axons respond differently to cells of the optic chiasm midline in vitro. *Neuron* 15, 1349-1364. 10.1016/0896-6273(95)90013-6 8845158

[DEV202446C49] Wang, L.-C., Rachel, R. A., Marcus, R. C. and Mason, C. A. (1996). Chemosuppression of retinal axon growth by the mouse optic chiasm. *Neuron* 17, 849-862. 10.1016/S0896-6273(00)80217-2 8938118

[DEV202446C50] Wang, Y., Li, G., Stanco, A., Long, J. E., Crawford, D., Potter, G. B., Pleasure, S. J., Behrens, T. and Rubenstein, J. L. R. (2011). CXCR4 and CXCR7 have distinct functions in regulating interneuron migration. *Neuron* 69, 61-76. 10.1016/j.neuron.2010.12.005 21220099 PMC3025760

[DEV202446C51] Williams, S. E., Mann, F., Erskine, L., Sakurai, T., Wei, S., Rossi, D. J., Gale, N. W., Holt, C. E., Mason, C. A. and Henkemeyer, M. (2003). Ephrin-B2 and EphB1 mediate retinal axon divergence at the optic chiasm. *Neuron* 39, 919-935. 10.1016/j.neuron.2003.08.017 12971893

[DEV202446C52] Xie, L., Cen, L.-P., Li, Y., Gilbert, H.-Y., Strelko, O., Berlinicke, C., Stavarache, M. A., Ma, M., Wang, Y., Cui, Q. et al. (2022). Monocyte-derived SDF1 supports optic nerve regeneration and alters retinal ganglion cells’ response to Pten deletion. *Proc. Natl. Acad. Sci. USA* 119, e2113751119. 10.1073/pnas.2113751119 35394873 PMC9169637

[DEV202446C53] Zhu, Y., Yu, T., Zhang, X.-C., Nagasawa, T., Wu, J. Y. and Rao, Y. (2002). Role of the chemokine SDF-1 as the meningeal attractant for embryonic cerebellar neurons. *Nat. Neurosci.* 5, 719-720. 10.1038/nn881 12080344 PMC2072873

[DEV202446C54] Zhu, Y., Matsumoto, T., Mikami, S., Nagasawa, T. and Murakami, F. (2009). SDF1/CXCR4 signalling regulates two distinct processes of precerebellar neuronal migration and its depletion leads to abnormal pontine nuclei formation. *Development* 136, 1919-1928. 10.1242/dev.032276 19429788

