# Supplementary material for: CXCL12 promotes the crossing of retinal ganglion cell axons at the optic chiasm
Source: Development. 2024 Jan 12;151(2):dev202446. doi: 10.1242/dev.202446 (PMC10820821; doi:10.1242/dev.202446)
Supplement: Supplementary information [file develop-151-202446-s1.pdf]

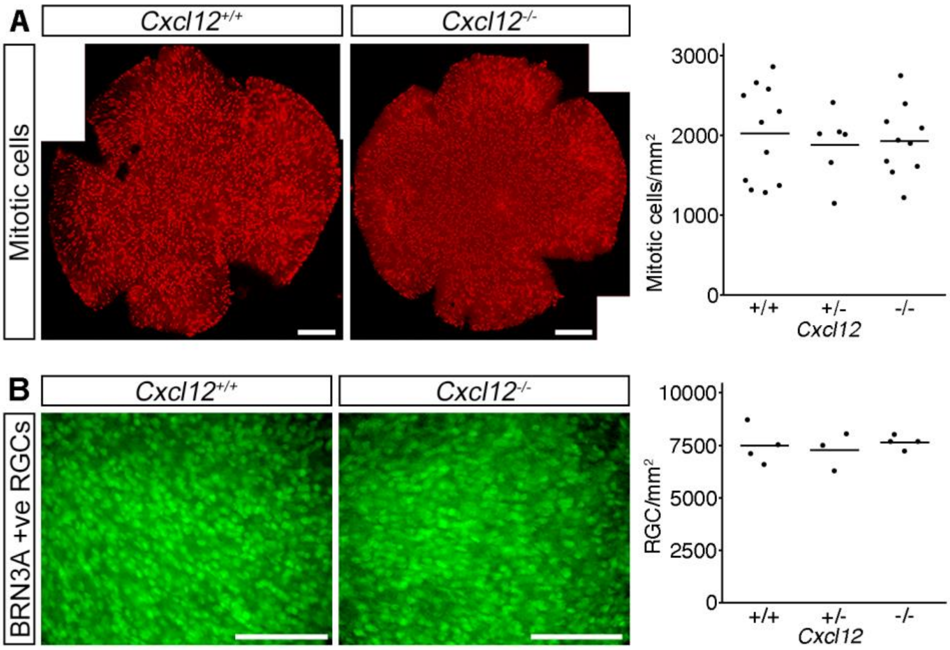

Fig. S1. Retinal development appears normal in E14.5 *Cxcl12* mutants.

(A-B) Representative examples and quantification of E14.5 *Cxcl12* wild-type and mutant flatmounted retinas stained with antibodies against phosphohistone H3 to label mitotic cells (A) or BRN3A to label RGCs (B). Panels in (A) are composite images generated by overlaying in Adobe Photoshop pictures captured from disparate retinal regions. Quantitative data are shown as the average of the mean, indicated with a horizontal bar; each dot represents the value for one embryo. Number of retinas analysed: phosphohistone H3, *Cxcl12*$^{+/+}$ n = 11, *Cxcl12*$^{+/-}$ n = 6, *Cxcl12*$^{-/-}$ n = 10; BRN3A, *Cxcl12*$^{+/+}$ n = 4, *Cxcl12*$^{+/-}$ n = 3, *Cxcl12*$^{-/-}$ n = 4. Scale bars, 200 µm (A), 100 µm (B).

Development • Supplementary information

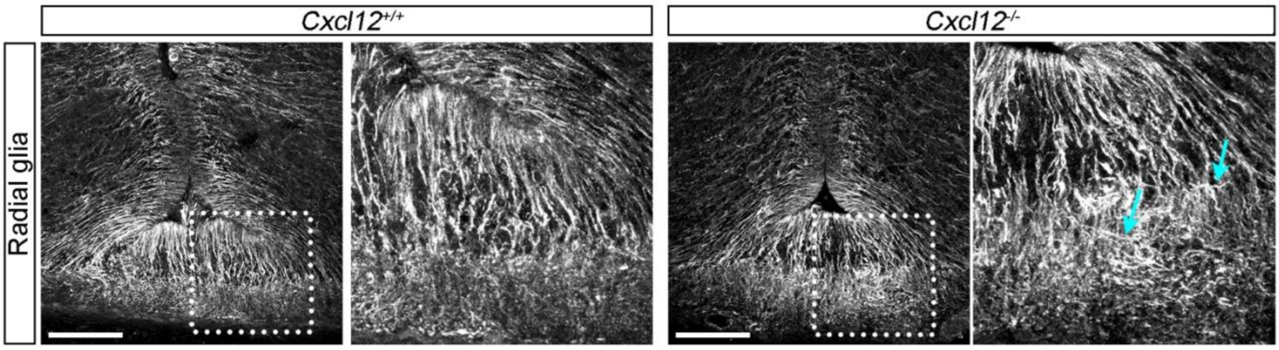

Fig. S2. Midline morphology appears relatively normal in E14.5 *Cxcl12* mutants

RC2 labelled radial glia at the E14.5 *Cxcl12*$^{+/+}$ and *Cxcl12*$^{-/-}$ ventral diencephalic midline (n = 3 each). Boxed regions are shown at higher magnification in the right panels. Blue arrows indicate example glial processes deviating from their normal radial trajectory. Scale bars, 100 µm.
