# Supplementary material for: CXCL12 promotes the crossing of retinal ganglion cell axons at the optic chiasm
Source: Development. 2024 Jan 12;151(2):dev202446. doi: 10.1242/dev.202446 (PMC10820821; doi:10.1242/dev.202446)
Supplement: Supplementary file 1 [file develop-151-202446_review_history.pdf]

CXCL12 promotes the crossing of retinal ganglion cell axons at the optic chiasm

Viet-Hang Le, Clarisse Orniacki, Verónica Murcia-Belmonte, Laura Denti, Dagmar Schütz, Ralf Stumm, Christiana Ruhrberg and Lynda Erskine
DOI: 10.1242/dev.202446

Editor: Francois Guillemot

Review timeline

Original submission:	20 October 2023
Editorial decision:	27 November 2023
First revision received:	6 December 2023
Accepted:	7 December 2023

Original submission

First decision letter

MS ID#: DEVELOP/2023/202446

MS TITLE: CXCL12 promotes the crossing of retinal ganglion cell axons at the optic chiasm.

AUTHORS: Viet-Hang Le, Clarisse Orniacki, Veronica Murcia-Belmonte, Laura Denti, Dagmar Schutz, Ralf Stumm, Christiana Ruhrberg, and Lynda Erskine

I have now received the reports of three referees on your manuscript and I have reached a decision. The referees' comments are appended below, or you can access them online: please go to BenchPress and click on the 'Manuscripts with Decisions' queue in the Author Area.

As you will see, all the referees are enthusiastic about your work, and we would like to publish a revised manuscript in Development, provided that the referees' comments can be satisfactorily addressed. Please attend to all of the reviewers' comments in your revised manuscript and detail them in your point-by-point response. If you do not agree with any of their criticisms or suggestions explain clearly why this is so. If it would be helpful, you are welcome to contact us to discuss your revision in greater detail. Please send us a point-by-point response indicating your plans for addressing the referees' comments, and we will look over this and provide further guidance.

Reviewer 1

Advance summary and potential significance to field

Most optic axons in mice cross to the contralateral side of the brain, while a minority remains ipsilateral. The exact molecular mechanisms by which the crossing/non crossing is achieved are still not fully known. Using mutant mice and chick retinal explants, the present paper determines that the cytokine CXCL12 through its receptor CXCR4, but not its alternative receptor ACKR3, stimulates axonal growth, which directs axons into the contralateral path. This indicates that, as in situations in which axons have to make a choice of direction, the time the axons interact with guidance molecules plays a major role.

© 2024. Published by The Company of Biologists under the terms of the Creative Commons Attribution License (https://creativecommons.org/licenses/by/4.0/).

Comments for the author

This is a brief and straightforward study, it is appropriately powered, beautifully illustrated and cautiously interpreted and discussed. I have only one minor point. In line 115 it should read "... THE NUMBER OF ipsilaterally projecting…"

Reviewer 2

Advance summary and potential significance to field

This paper points to influences of growth support by the meninges, 'outlining' the tracts of retinal ganglion cell axons from eye to brain. A major factor identified in the meninges is SDF (CXL12). The study highlights a new guidance mechanism that many act in parallel , or be accessory to, the well known factors acting in chiasm formation. It should be welcome to the field.

Comments for the author

Le et al., from the lab of Lynda Erskine, address how CXCL 12 (SDF1), expressed in the meninges along the pathway from eye to the brain, aids in the progression of retinal ganglion cell (RGC) axons as the segregate at the midline of the ventral diencephalon during optic chiasm formation. All retinal ganglion cells, both ipsilaterally- and contralaterally-projecting, express the receptor to CXCL12 - CXCR4 - but in the knockout of CXCL12 or CXCR4, there is an increase RGCs that project ipsilaterally primarily from cells outside of the VTC, home to the ipsilateral RGCs. These authors go on to propose that meningeal-derived CXCL12 aids, especially for contralateral RGCs, growth at the midline. This study is welcome because it adds I new dimension to the factors guiding retinal axon decussation.

The experiments are very well done, the data succinctly presented, and the writing clear for the most part. Most of my suggested revisions are textual.

Major revisions/questions:
1. The case for CXCL12 being important for the contralateral cells is strong, but less so for the ipsilateral RGCs emanating from the VTC:
a. ll 11-15 become clear only after having read the manuscript. As is, these lines do not intimate their conclusion that CXCL12 is important for contralateral growth. Perhaps citing in ll 7-8 that the increase in RGCs projecting contralaterally occurs primarily from elsewhere in the retina, as well as the VTC (see a. above). or, add ll 120-121 to the abstract in some form.
b. RGCs that project ipsilaterally in the mutants: ll 111-116: Is there a decrease in the number of cells in VTC retina that project ipsilaterally? Do they project contralaterally?? A box could be placed on the VTC on the flatmounts in Figure 2.B to make this aspect clearer
c. ll 190-196 focuses on the contralaterally-projecting RGC axons. The authors propose that CXCL12 might dampen the response of (contralateral) RGC axons to inhibitory signals. Again, what about the ipsi's? And might the CXCL12 normally interact (instead of dampening, rather, fortifying…) with the ipsi cues at the midline such as the ephrins?

2. ll 162-163 and Figure 4B: "Outgrowth of both ipsilaterally and contralaterally projecting RGC axons also was increased significantly in the presence of ventral diencephalon meninges". It appears as though the retinal axons were touching (barely) the meninges. Please describe the culture setup a bit more: If in Matrigel, and the meninges places opposite the explant, the expectation is that CXCL12 would be secreted, mimicking adding CXCL12 to Matrigel. But in vivo, the axons are growing on the meninges. Please explain this discrepancy.
3. L 61 - "…enabling axon growth towards the chiasm midline": true of both ipsi- and contralaterally coursing axons?
4. Figure 3A - are the "radially oriented cells" in the retina Mueller glia?
5. ll 133-135 on the midline glia, with some of their fibers less oriented and appear "free floating. Is CXCL12 needed for endfeet attachment of the midline glia?
6. ll. 55-56: "…it is not known whether meninges-derived CHCL12 or its receptors CXCR4 and ACKR3 are essential for optic pathway development", yet on ll 165-166, a note is made that CXCL12 neither collapses or attracts check RGCs, indicating that others have studied RGCs and the role of

© 2024. Published by The Company of Biologists under the terms of the Creative Commons Attribution License (https://creativecommons.org/licenses/by/4.0/).

CXCL12, and then in the next paragraph, the studies of Chalasani are cited on interactions with Slit. These studies are interesting but came as a surprise because of the sentence on ll 55-56. Perhaps in the latter lines, mention could be made that SDF has been studied in RGCs but not n its role in decussation.

Additional minor comments:
1. L 19 - RGC should be RGCs
2. L 22 - “meet at the optic chiasm” should be “meet to form the optic chiasm”
3. L 199: a newer reference on regeneration by Varadarajan and Huberman is PMID 34995518.

Reviewer 3

Advance summary and potential significance to field

The significance of this study is that it identifies a new cue/receptor guidance system for retinal ganglion axons in developing mouse embryos. Retinal ganglion axons project to the optic chiasm and reach this decision point to either grow across the chiasm to the contralateral diencephalon, or instead to grow into the ipsilateral side. Evidence is presented that the CXCL12/CXCR4 ligand/receptor signaling system contributes an important signal to promote the growth of axons at the chiasm. The secreted ligand is expressed by a layer of meningeal cells next to the chiasm, and the CXCR4 receptor is expressed in the retinal ganglion neuron layer of the developing retina. The key finding is that CXCL12 mutant mouse embryos have an increased number of axons that turn away from the chiasm to grow ipsilaterally, and similar axon errors are seen in CXCR4 mutant embryos.
An alternative receptor expressed in the retina does not have this type of error. The study also tests the direct effect of CXCL12 on cultured retinal explants, showing that this signal increases axon growth. Together, these findings are convincing and well-documented to support the main conclusion of the paper. The significance is adding CXCL12/CXCR4 signaling to a list of other guidance cues for retinal axons at the chiasm. Whether and how this new signal interacts with the previously identified cues is not addressed by the experiments presented, which would have added to the significance. For example the growth promoting signal would be predicted to balance against other repellent or inhibitory signals, which could be tested by combinations of signals in axon cultures. A broader significance of a new growth promoting signal would be as a potential signal to promote retinal axon regeneration.

Comments for the author

Revisions suggested:
1. In the analysis of the CXCL12 mutants, the increased ipsilateral projects are described as defasciculated. While they do appear defasciculated from the normal ipsilateral tract, the extra projects also do appear aligned with the expected position of the broader contralateral tract. It therefore seems likely that the extra ipsilateral axons are fasciculated with contralateral axons, and so are not necessarily mis-guided but still following the expected optic tract.
2. Add more detailed description about the image collected of the optic tracts. If these images were collected on a confocal microscope, and presented as a Z-stack projection, that would be important information to know. Related to this, was the quantification done on the Z image stacks, or on the single projected image?
3. Add age information to either Figure 2 or to the legend, regarding embryo age. Please clarify whether the retrograde labeling from the thalamus was done at the same age?
4. Not a required revision, but the explant cultures could have reached a stronger conclusion if CXCL12 was added in combination with other cues, for example Slit inhibitory cues, to test for cue interactions such as “dampens”.
That could potentially add evidence to the working model presented in the last paragraph.
5. Line 189: “finds” should be “findings”?.

© 2024. Published by The Company of Biologists under the terms of the Creative Commons Attribution License (https://creativecommons.org/licenses/by/4.0/).

First revision

Author response to reviewers' comments

Response to Reviewer 1

Reviewer 1 Advance Summary and Potential Significance to Field:
Most optic axons in mice cross to the contralateral side of the brain, while a minority remains ipsilateral. The exact molecular mechanisms by which the crossing/non crossing is achieved are still not fully known. Using mutant mice and chick retinal explants, the present paper determines that the cytokine CXCL12 through its receptor CXCR4, but not its alternative receptor ACKR3, stimulates axonal growth, which directs axons into the contralateral path. This indicates that, as in situations in which axons have to make a choice of direction, the time the axons interact with guidance molecules plays a major role.

Reviewer 1 Comments for the Author:
This is a brief and straightforward study, it is appropriately powered, beautifully illustrated and cautiously interpreted and discussed.

I have only one minor point. In line 115 it should read "... THE NUMBER OF ipsilaterally projecting…"
REPLY: We have now corrected the error in the text.

*****
Response to Reviewer 2

Reviewer 2 Advance Summary and Potential Significance to Field:
This paper points to influences of growth support by the meninges, 'outlining' the tracts of retinal ganglion cell axons from eye to brain. A major factor identified in the meninges is SDF (CXL12). The study highlights a new guidance mechanism that many act in parallel , or be accessory to, the well known factors acting in chiasm formation. It should be welcome to the field.
REPLY: We thank the Reviewer for highlighting the value of our study for the field.

Reviewer 2 Comments for the Author:
Le et al., from the lab of Lynda Erskine, address how CXCL 12 (SDF1), expressed in the meninges along the pathway from eye to the brain, aids in the progression of retinal ganglion cell (RGC) axons as the segregate at the midline of the ventral diencephalon during optic chiasm formation. All retinal ganglion cells, both ipsilaterally- and contralaterally-projecting, express the receptor to CXCL12 - CXCR4 - but in the knockout of CXCL12 or CXCR4, there is an increase RGCs that project ipsilaterally primarily from cells outside of the VTC, home to the ipsilateral RGCs. These authors go on to propose that meningeal-derived CXCL12 aids, especially for contralateral RGCs, growth at the midline. This study is welcome because it adds I new dimension to the factors guiding retinal axon decussation. The experiments are very well done, the data succinctly presented, and the writing clear for the most part. Most of my suggested revisions are textual.
REPLY: We thank the Reviewer for the accurate summary and are grateful that our data are well received. We will revise the text as recommended below.

Major revisions/questions:
1. The case for CXCL12 being important for the contralateral cells is strong, but less so for the ipsilateral RGCs emanating from the VTC:
a.ll 11-15 become clear only after having read the manuscript. As is, these lines do not intimate their conclusion that CXCL12 is important for contralateral growth. Perhaps citing in ll 7-8 that the increase in RGCs projecting contralaterally occurs primarily from elsewhere in the retina, as well as the VTC (see a. above). or, add ll 120-121 to the abstract in some form.
REPLY: We have added a statement to the abstract that the increased proportion of ipsilaterally projecting RGC is due to misrouting of presumptive contralaterally-specified RGCs.

b.RGCs that project ipsilaterally in the mutants: ll 111-116: Is there a decrease in the number of

© 2024. Published by The Company of Biologists under the terms of the Creative Commons Attribution License (https://creativecommons.org/licenses/by/4.0/).

cells in VTC retina that project ipsilaterally? Do they project contralaterally?? A box could be placed on the VTC on the flatmounts in Figure 2.B to make this aspect clearer
REPLY: The graphs in Figure 2B have been replotted to show the proportion of ipsilaterally projecting RGCs relative to the total number of labelled RGCs in both eyes both within and outside the VTC. This analysis demonstrates that the *Cxcl12* and *Cxcr4* mutations have no impact on the ipsilateral projection arising from the VTC, whereas the number of ipsilaterally projecting RGCs located outside the VTC is increased significantly. These findings have now been discussed (pages 5 and 6 of the revised manuscript).

c.ll 190-196 focuses on the contralaterally-projecting RGC axons. The authors propose that CXCL12 might dampen the response of (contralateral) RGC axons to inhibitory signals. Again, what about the ipsi's? And might the CXCL12 normally interact (instead of dampening, rather, fortifying…) with the ipsi cues at the midline such as the ephrins?
REPLY: In vivo, loss of CXCL12 has no obvious impact on RGC axons that emerge from the VT retina and project ipsilaterally (see new Figure 2B). We have modified our proposed model to discuss specifically why we believe routing of ipsilaterally projecting axons is not altered in the absence of CXCL12 signalling (pages 7 and 8 of revised manuscript).

2. ll 162-163 and Figure 4B: "Outgrowth of both ipsilaterally and contralaterally projecting RGC axons also was increased significantly in the presence of ventral diencephalon meninges". It appears as though the retinal axons were touching (barely) the meninges. Please describe the culture setup a bit more: If in Matrigel, and the meninges places opposite the explant, the expectation is that CXCL12 would be secreted, mimicking adding CXCL12 to Matrigel. But in vivo, the axons are growing on the meninges. Please explain this discrepancy.
REPLY: In vivo, RGCs axons grow in close proximity to the meninges, and we assume that some may be in direct contact whilst others would be further away, given the width of the optic chiasm. As we expect that CXCL12 would be secreted from the meningeal tissue, the axon distance should not matter, as long as they are fairly close, within the SDF range. With these considerations in mind, we cultured retinal explants in collagen gels at a short distance (100-400 µm) from the meninges tissue; we have now clarified this (page 7 of the revised manuscript).

3. L 61 - "…enabling axon growth towards the chiasm midline": true of both ipsi- and contralaterally coursing axons?
REPLY: Our data support that growth of both ipsilaterally and contralaterally projecting RGC axons towards the midline will be impaired. We propose that routing of ipsilaterally axons is not altered in the absence of CXCL12 because repulsion from the midline is essential for their guidance into the ipsilateral optic tract. We have expanded the final section of the Results and Discussion section to better explain why we believe the routing of contralaterally-projecting axons is selectively affected in the absence of CXCL12 signalling, despite CXCL12 being growth promoting for all RGC axons (pages 7 and 8 of revised manuscript).

4. Figure 3A - are the "radially oriented cells" in the retina Mueller glia?
REPLY: The radially oriented cells were detected at E12.5 and E14.5, whereas Müller glia are generated predominately postnatally in rodents (Anat Rec 212, 199; J Comp Neurol 474, 304). It therefore is unlikely that these radially oriented cells are Müller glia. Instead, these cells may be nascent RGCs that have not yet retracted their apical process and translocated into the RGC layer, subsequent to the cell cycle-associated interkinetic nuclear migration of their progenitors (Page 6 of revised manuscript).

5. ll 133-135 on the midline glia, with some of their fibers less oriented and appear "free floating. Is CXCL12 needed for endfeet attachment of the midline glia?
REPLY: Indeed, it has been shown for the spinal cord that CXCL12 is important for endfeet attachment of midline glia. We have now added a sentence to highlight this prior work and to raise the possibility that this role extends to the ventral diencephalon (page 6 of revised manuscript).

6. ll. 55-56: "…it is not known whether meninges-derived CXCL12 or its receptors CXCR4 and ACKR3 are essential for optic pathway development", yet on ll 165-166, a note is made that CXCL12 neither collapses or attracts chick RGCs, indicating that others have studied RGCs and the role of CXCL12, and then in the next paragraph, the studies of Chalasani are cited on

© 2024. Published by The Company of Biologists under the terms of the Creative Commons Attribution License (https://creativecommons.org/licenses/by/4.0/).

interactions with Slit. These studies are interesting but came as a surprise because of the sentence on ll 55-56. Perhaps in the latter lines, mention could be made that SDF has been studied in RGCs but not n its role in decussation.
REPLY: We have clarified in the introduction that CXCL12 signalling has been shown to modulate the RGC axon response to inhibitory guidance signals in vivo and in vitro, but a role in RGC axon segregation at the optic chiasm has not previously been investigated (page 4 of revised manuscript).

Additional minor comments:
1.L 19 - RGC should be RGCs
2.L 22 - “meet at the optic chiasm” should be “meet to form the optic chiasm”
3.L 199: a newer reference on regeneration by Varadarajan and Huberman is PMID 34995518.
REPLY: We have made these three changes.

*****
Response to Reviewer 3

Reviewer 3 Advance Summary and Potential Significance to Field:
The significance of this study is that it identifies a new cue/receptor guidance system for retinal ganglion axons in developing mouse embryos. Retinal ganglion axons project to the optic chiasm and reach this decision point to either grow across the chiasm to the contralateral diencephalon, or instead to grow into the ipsilateral side. Evidence is presented that the CXCL12/CXCR4 ligand/receptor signaling system contributes an important signal to promote the growth of axons at the chiasm. The secreted ligand is expressed by a layer of meningeal cells next to the chiasm, and the CXCR4 receptor is expressed in the retinal ganglion neuron layer of the developing retina. The key finding is that CXCL12 mutant mouse embryos have an increased number of axons that turn away from the chiasm to grow ipsilaterally, and similar axon errors are seen in CXCR4 mutant embryos. An alternative receptor expressed in the retina does not have this type of error. The study also tests the direct effect of CXCL12 on cultured retinal explants, showing that this signal increases axon growth. Together, these findings are convincing and well- documented to support the main conclusion of the paper.
REPLY: We thank the Reviewer for their accurate summary that places our study into context of the field, and we are pleased that the Reviewer finds our data convincing.

The significance is adding CXCL12/CXCR4 signaling to a list of other guidance cues for retinal axons at the chiasm. Whether and how this new signal interacts with the previously identified cues is not addressed by the experiments presented, which would have added to the significance. For example, the growth promoting signal would be predicted to balance against other repellent or inhibitory signals, which could be tested by combinations of signals in axon cultures. A broader significance of a new growth promoting signal would be as a potential signal to promote retinal axon regeneration.
REPLY: We agree with the reviewer that our study opens the door to further experiment to explore interaction with other relevant signals. Whilst we acknowledge that this has not yet been done, we have submitted the current brief but definitive report, in the hope that others will be able to draw on it to provide further detail on exactly those pathway interactions.

Reviewer 3 Comments for the Author:
Revisions suggested:

1. In the analysis of the CXCL12 mutants, the increased ipsilateral projects are described as defasciculated. While they do appear defasciculated from the normal ipsilateral tract, the extra projects also do appear aligned with the expected position of the broader contralateral tract. It therefore seems likely that the extra ipsilateral axons are fasciculated with contralateral axons, and so are not necessarily mis-guided but still following the expected optic tract.
REPLY: We have modified our description to remove reference to fasciculation and instead now state that the ipsilateral axons occupy a broader domain in the mutants. We have added a sentence to explain that this may be because the misrouted contralaterally- fated RGC axons aligns with contralateral rather than ipsilateral axons from the other eye (page 5 of revised manuscript).

2. Add more detailed description about the image collected of the optic tracts. If these images were collected on a confocal microscope, and presented as a Z-stack projection, that would be

© 2024. Published by The Company of Biologists under the terms of the Creative Commons Attribution License (https://creativecommons.org/licenses/by/4.0/).

important information to know. Related to this, was the quantification done on the Z image stacks, or on the single projected image?
REPLY: Images were collected using a stereo microscope, not a confocal. This has now been explained in the legend for Figure 2 (page 17 of revised manuscript).

3. Add age information to either Figure 2 or to the legend, regarding embryo age.
REPLY: Embryo age has been included in the legend for Figure 2.

Please clarify whether the retrograde labeling from the thalamus was done at the same age?
REPLY: All retrograde labelling was performed on E15.5 littermates, whereas anterograde analyses were performed on E14.5 littermates. Anterograde labelling at E14.5 will label axons from all RGCs generated at this stage, but many of their axons will not have reached the dorsal thalamus. We therefore performed the retrograde labelling at E15.5, when more of the axons would have reached the dorsal thalamus

4. Not a required revision, but the explant cultures could have reached a stronger conclusion if CXCL12 was added in combination with other cues, for example Slit inhibitory cues, to test for cue interactions such as “dampens”. That could potentially add evidence to the working model presented in the last paragraph.
REPLY: Previous work using chicken retinal explants has demonstrated that CXCL12 can ameliorate the inhibitory effect of Slits on RGC growth cone collapse and axon outgrowth. We have now cited this prior work in the Introduction (page 4 of revised manuscript).

5. Line 189: “finds” should be “findings”?.
REPLY: Yes, it should be, and we have corrected this.

<u>Second decision letter</u>

MS ID#: DEVELOP/2023/202446

MS TITLE: CXCL12 promotes the crossing of retinal ganglion cell axons at the optic chiasm.

AUTHORS: Viet-Hang Le, Clarisse Orniacki, Veronica Murcia-Belmonte, Laura Denti, Dagmar Schutz, Ralf Stumm, Christiana Ruhrberg, and Lynda Erskine
ARTICLE TYPE: Research Report

I am delighted to tell you that your manuscript has been accepted for publication in Development, pending our standard ethics checks.

© 2024. Published by The Company of Biologists under the terms of the Creative Commons Attribution License (https://creativecommons.org/licenses/by/4.0/).
